# Prevalence of low-level viremia in people living with HIV/AIDS and its impact on virological failure in Guizhou Province, China

**DOI:** 10.3389/fpubh.2025.1535285

**Published:** 2025-07-30

**Authors:** Yan Zheng, Xinling Zha, Hai Long, Wenji Zeng, Junhua Wang, Yongming Yao, Lu Huang, Xiaotian Song, Maosi Wang, Yang Chen

**Affiliations:** ^1^Guiyang Public Health Clinical Center, Guiyang, China; ^2^School of Public Health, The Key Laboratory of Environmental Pollution Monitoring and Disease Control, Ministry of Education, Guizhou Medical University, Guiyang, China; ^3^Guizhou Center For Disease Control And Prevention, Guiyang, China; ^4^Guizhou Medical University, Guiyang, China

**Keywords:** AIDS, antiretroviral therapy, epidemiological characteristics, Guizhou Province, influencing factors, low-level viremia

## Abstract

**Background:**

The frequency of low-level viremia (LLV) may vary across China. This study aims to analyze the epidemiological characteristics and related factors of LLV among HIV/AIDS patients in Guizhou Province from 2016 to 2022, and further analyze the impact of LLV on virological failure (VF).

**Research design and methods:**

In this retrospective cohort study, we analyzed people living with HIV/AIDS whose demographic data, CD4^+^ T lymphocyte count, and viral load. LLV was defined as at least one VL measurement between 50 and 999 copies/mL after ART. Viral suppression refers to the maintenance of a VL < 50 copies/mL after undergoing ART. VF was defined as a VL ≥ 1,000 copies/mL after ART. To examine and compare the incidence of LLV at different levels, this study divided LLV into three groups based on previous research definitions: low-level LLV (LLLV) ranging from 50 to 199 copies/mL, medium-level LLV (MLLV) ranging from 200 to 399 copies/mL, and high-level LLV (HLLV) ranging from 400 to 999 copies/mL. LLV occurrence frequency was divided into two groups: intermittent LLV (iLLV) was defined as one independent LLV with both the previous and the subsequent viral load below the lower limit of detection. Persistent LLV (pLLV) was defined as at least two consecutive episodes of LLV. We divided the people living with HIV/AIDS into two sub-cohorts based on whether they experienced LLV. Non-LLV was defined as never having experienced LLV. We compared the occurrence of LLV at different levels and frequencies, as well as the risk of virological failure (VF), using the Chi-square test. Non-conditional binary logistic regression analysis was used to identify factors influencing LLV.

**Results:**

In total, 28,613 cases of infection were analyzed, with 33.72% (9,649/28,613) exhibiting LLV. The LLV proportion rates were 20.69, 6.50, and 6.48% in the low-level LLV (LLLV) (50–199 copies/mL), medium-level LLV (MLLV) (200–399 copies/mL), and high-level LLV (HLLV) (400–999 copies/mL) groups, respectively. The intermittent LLV (iLLV) and persistent LLV (pLLV) groups had LLV rates of 28.65 and 5.07%, respectively. The VF rates were 6.18, 11.79, and 13.70% in the LLLV, MLLV, and HLLV groups, respectively (*p* < 0.001). The iLLV and pLLV groups had VF rates of 8.82 and 8.14%, respectively (*p* = 0.397). Multivariate logistic regression analysis revealed that the gender, ethnicity, age at antiretroviral therapy initiation, baseline CD4^+^ T lymphocyte count, and current treatment regimen were factors influencing the occurrence of LLV.

**Conclusion:**

The incidence of LLV among people living with HIV/AIDS treated with INSTI-sparing regimens in Guizhou Province is relatively high and is mainly influenced by factors such as age, CD4^+^ T lymphocyte count and treatment regimens. A comprehensive assessment of these factors is essential to implement targeted interventions that prevent LLV and enhance the effectiveness of antiretroviral therapy.

## 1 Introduction

Acquired immunodeficiency syndrome (AIDS) is a chronic infectious disease and a major global public health concern (1). Despite the lack of an effective vaccine for prevention, antiretroviral therapy (ART) can effectively suppress viral replication, resulting in a reduction of the viral load (VL) in the peripheral blood of most people living with HIV/AIDS (PLWHA) to undetectable levels (2). Low-level viremia (LLV) is defined as a viral load between 50 and 1,000 copies/ml according to the World Health Organization (WHO) guidelines (3). Studies have shown that even after long-term standardized treatment, a small number of PLWHA may still develop low-level viremia (LLV), where LLV that occurs only once or several times at intervals is called intermittent LLV (iLLV), and LLV that occurs 2 or more times in a row is called persistent LLV (pLLV) (4, 5). As per the “Undetectable equals Untransmittable (U=U)” paradigm, PLWHA with LLV still carry the risk of transmitting the virus (6). Furthermore, persistent viral replication can result in drug resistance and increase the likelihood of virological failure (VF) and death (7).

Existing LLV studies have mainly been conducted in developed countries in Europe and North America (8–10) rather than in low- and middle-income countries or regions. In South Africa, evidence from a multicenter observational cohort study that followed World Health Organization (WHO) definitions indicated that an LLV of 51–999 copies/mL was associated with subsequent VF, which can be viewed as an early warning sign of VF. The authors of the study recommended that clinical practitioners in low-and middle-income countries and regions following the WHO guidelines should strengthen their efforts to differentiate and manage LLV properly, and policymakers should include recommendations for the clinical management of LLV in the WHO guidelines and reconsider a lower threshold for VF.

Guizhou Province, located in southwest China, is home to a diverse population, including many ethnic minorities. The region remains relatively underdeveloped economically. In 2023, its gross domestic product (GDP) was 2.09 trillion yuan, accounting for only 44.28% of that of Shanghai, a more developed province. The first HIV infection in Guizhou was reported in 1993. Since then, the epidemic has spread annually; it has currently affected all 9 prefectures and 88 counties (districts) in the province. Cases span over 30 ethnic groups, making Guizhou one of the most severely affected regions by AIDS in China.

Before 2016, ART and follow-up care were primarily managed by Centers for Disease Control and Prevention (CDC). After 2016, the responsibility for ART services in all 88 counties was transferred to designated hospitals. Guizhou has since implemented a three-tier ART service delivery model. The primary medical institutions (e.g., township health centers and community health service centers) are responsible for pilot drug distribution and follow-up management. Secondary institutions (county-level hospitals) provide most routine treatment, follow-up, and drug dispensing. Tertiary institutions (provincial or municipal hospitals) handle complex cases—such as drug resistance or opportunistic infections—and offer technical guidance and training.

Currently, most PLWHA in Guizhou Province receive free ART, as recommended by National HIV/AIDS free antiretroviral therapy manual of China. However, although the PLWHA have received ART since 2005, no studies have been conducted on LLV during nearly two decades of ART. Therefore, this study examined the frequency of LLV among PLWHA undergoing ART in Guizhou Province between 2016 and 2022. The objective of this study was to gain insight into the occurrence of LLV in Guizhou Province and identify the factors that contribute to its prevalence. The findings of this study will provide valuable scientific evidence for reducing the risk of HIV transmission, enhancing the quality of life of PLWHA, and developing effective prevention and control strategies.

## 2 Methods

### 2.1 Study population

From January 1, 2016 to December 31, 2022, PLWHA underwent ART in HIV/AIDS treatment facilities at all levels in Guizhou Province. The inclusion criteria were: (1) age ≥ 18 years; (2) ART treatment duration ≥ 6 months; and (3) at least one VL record. The exclusion criteria were: (1) an initial VL test result ≥1,000 copies/mL after 6 months of treatment; (2) During the follow-up observation period, VF occurred before LLV; and (3) no baseline CD4^+^ T lymphocyte test results were available. PLWHA were categorized into two groups: those with LLV and those without LLV. Ten variables were considered: gender, marital status, education level, ethnicity, mode of transmission, age at initiation of ART, time from diagnosis to treatment, baseline WHO clinical stage, baseline CD4^+^ T lymphocyte count, and current treatment regimen. The dependent variable was the VL outcome.

### 2.2 Source of data

To obtain data on the treatment status, follow-up records, and laboratory test results of PLWHA, we downloaded their medical history cards from the HIV/AIDS ART database of Guizhou Province. This retrospective cohort study began when the PLWHA commenced ART, with the observation period ending on December 31, 2022. The Primary outcome is the occurrence of LLV. According to the national policy for free ART, each PLWHA receive one VL test for free each calendar year, which means that PLWHA will be tested free for VL at a visit ≥6 months after ART initiation and repeat the VL test once for each subsequent year (11). In reality, in addition to the single VL test provided free by the national program, additional VL tests may be ordered at the discretion of HIV care providers at each site with the testing cost covered by patients out of pocket. Therefore, some PLWHA may have more than one documented VL result per year after receiving ART. In this study, the highest result was recorded in patients with multiple VL test results in the same year.

HIV viral load testing in Guizhou Province is performed by designated third-party testing institutions using the reverse transcription polymerase chain reaction method. These institutions are equipped with the necessary instruments, reagents, and technical capabilities. Nucleic acid extraction and amplification are conducted using fully automated systems. Each laboratory operates at least two detection platforms, with reagents having a sensitivity threshold of <50 copies/mL. In cases of uncertain results, retesting can be performed using multiple detection systems.

Current equipment includes systems from Roche, Zhuhai Lijuzhi, and Northeast Pharmaceutical. Upon receiving notification from the purchasing agency, AIDS treatment centers at the county and township levels collect samples within 24 h. Sample preservation and transportation are conducted in accordance with biosafety regulations. Testing is completed within 24 h of sample receipt by the laboratory. After testing, residual samples are either stored or disposed of following regulatory guidelines.

A remote-printed, encrypted, and officially stamped test report is issued within 48 h. Purchasers are provided with a dedicated username and password, and district- and county-level CDC can be granted management access upon request. Moreover, monthly test result databases are submitted to the AIDS Confirmation Center Laboratory of Guizhou Province. Tests used for research and epidemiological monitoring in the province are highly sensitive, with a detection limit of <50 copies/mL. Viral load test results below the detection limit or reported as “Target Not Detected” are recorded as 0. Quantifiable are recorded as numerical values.

### 2.3 Virological thresholds

Viral suppression refers to the maintenance of a VL < 50 copies/mL after undergoing ART. VF was defined as a VL ≥ 1,000 copies/mL after ART. LLV was defined as at least one VL measurement between 50 and 999 copies/mL after ART. If LLV occurred before VF during follow-up, it was also considered LLV occurrence. Currently, there is no unified standard for defining LLV (12). To examine and compare the incidence of LLV at different levels, this study divided LLV into three groups based on previous research definitions (13, 14): low-level LLV (LLLV) ranging from 50 to 199 copies/mL, medium-level LLV (MLLV) ranging from 200 to 399 copies/mL, and high-level LLV (HLLV) ranging from 400 to 999 copies/mL. For participants with multiple LLV records, the highest record was used for grouping. LLV occurrence frequency was divided into two groups: intermittent LLV (iLLV) was defined as one independent LLV with both the previous and the subsequent viral load below the lower limit of detection. Persistent LLV (pLLV) was defined as at least two consecutive episodes of LLV. Non-LLV was defined as never having experienced LLV. Presently, most patients in Guizhou province are treated according to Chinese antiviral therapy guidelines. The recommended first-line regimen includes one non-nucleoside reverse transcriptase inhibitor (NNRTI) combined with two nucleoside reverse transcriptase inhibitors (NRTIs). The preferred combination is TDF or AZT + 3TC + EFV or NVP. The second-line regimen is based on protease inhibitors (PIs) and includes two or three NRTIs combined with one PI. The recommended second-line regimen is TDF or AZT + 3TC + LPV/r. Other regimens are defined as those comprising one, two, or three drugs not included in the recommended first-line or second-line regimens.

### 2.4 Statistical methods

Excel 2016 was used to organize the data and SPSS 23.0 was used for statistical analysis. Data are shown as the median (*M*) and interquartile range (*IQR*) from the 25th to 75th percentile. Categorical variables are presented as frequencies and percentages. Comparisons between groups were made using the Chi-square test, and non-conditional binary logistic analysis was used to identify the factors that influenced the occurrence of LLV. A *p*-value less than 0.05 (two-sided test) was considered to indicate a statistically significant difference.

## 3 Results

### 3.1 Characteristics of study participants

**Figure 1:**
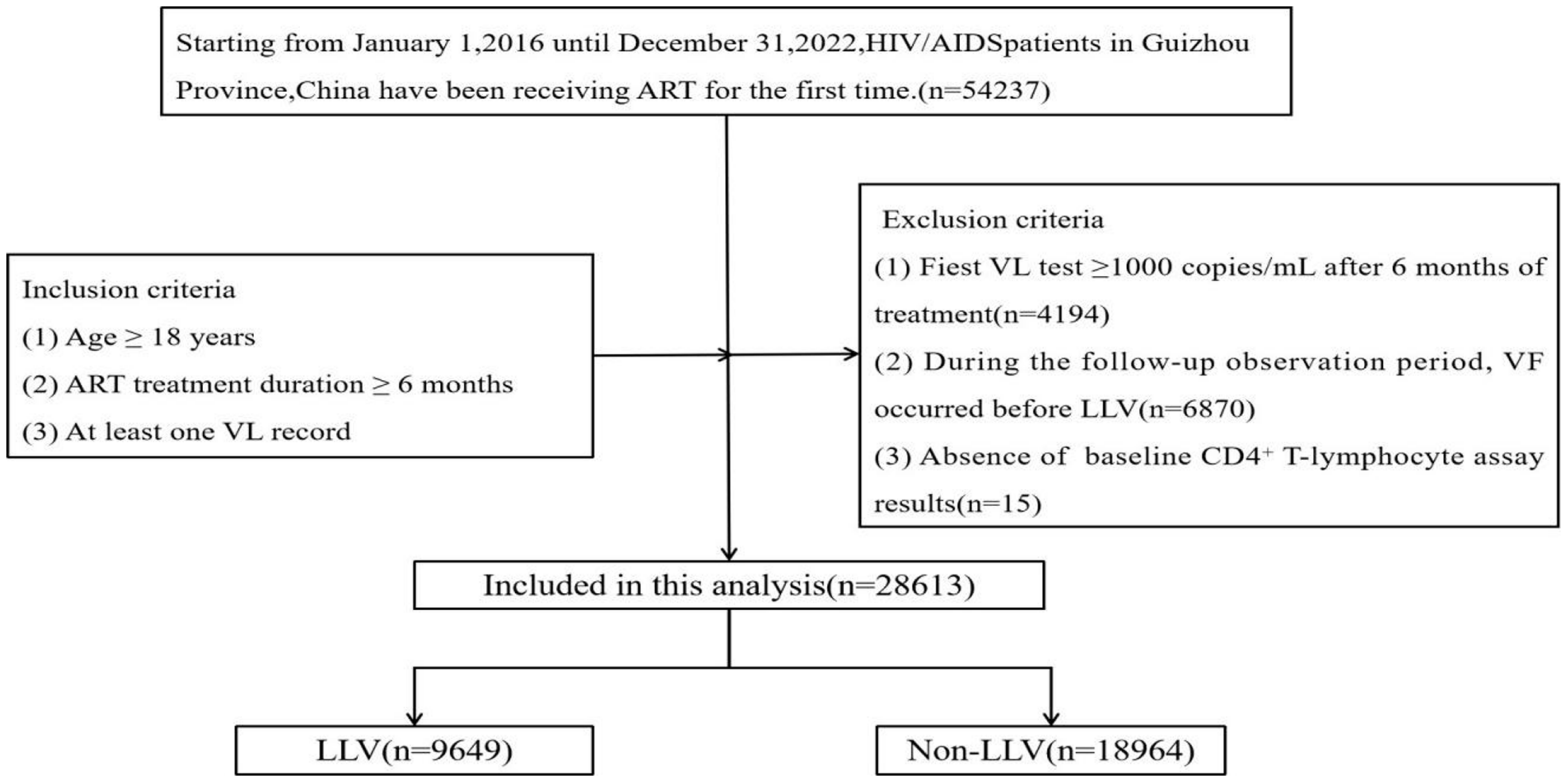
Flowchart showing selection of the participants. ART, antiretroviral therapy; HIV/AIDS, human immunodeficiency virus/acquired immunodeficiency syndrome; LLV, low-level viremia; VL, viral load.

In this study, a total of 28,613 PLWHA were included (Figure 1). Of the PLWHA, 65.58% (18,763/28,613) were male, 41.22% (11,794/28,613) were married, 38.50% (11,016/28,613) had completed primary school, and 71.08% (20,337/28,613) were of Han Chinese ethnicity. Heterosexual transmission was the most common route of transmission, accounting for 90.50% (25,895/28,613). Most PLWHA (46.19%, 13,216/28,613) were middle-aged or older (≥50 years) at the start of ART, and 89.11% (25,497/28,613) began treatment within 1 year of diagnosis. At baseline, 35.13% (10,052/28,613) had a CD4^+^ T-lymphocyte count of 200–350 cells/uL. The current treatment regimen for most PLWHA (84.85%, 24,279/28,613) was the first-line treatment regimen.

### 3.2 Occurrence of LLV

As of December 31, 2022, there were 9,649 cases of LLV, resulting in an incidence rate of 33.72% (9,649 out of 28,613). Of these cases, 5,920 were classified as LLLV, with an incidence rate of 20.69% (5,920 of 28,613); 1,874 were categorized as MLLV, with an incidence rate of 6.55% (1,874 of 28,613); and 1,854 were identified as HLLV, with an incidence rate of 6.48% (1,854 of 28,613). iLLV accounted for 28.65% (8,199 of 28,613) of the cases, while pLLV accounted for 5.07% (1,450 of 28,613). LLV did not occur in the remaining 66.28% of PLWHA (18,964/28,613).

### 3.3 Risk of VF in PLWHA with different levels and frequencies of LLV

**Figure 2:**
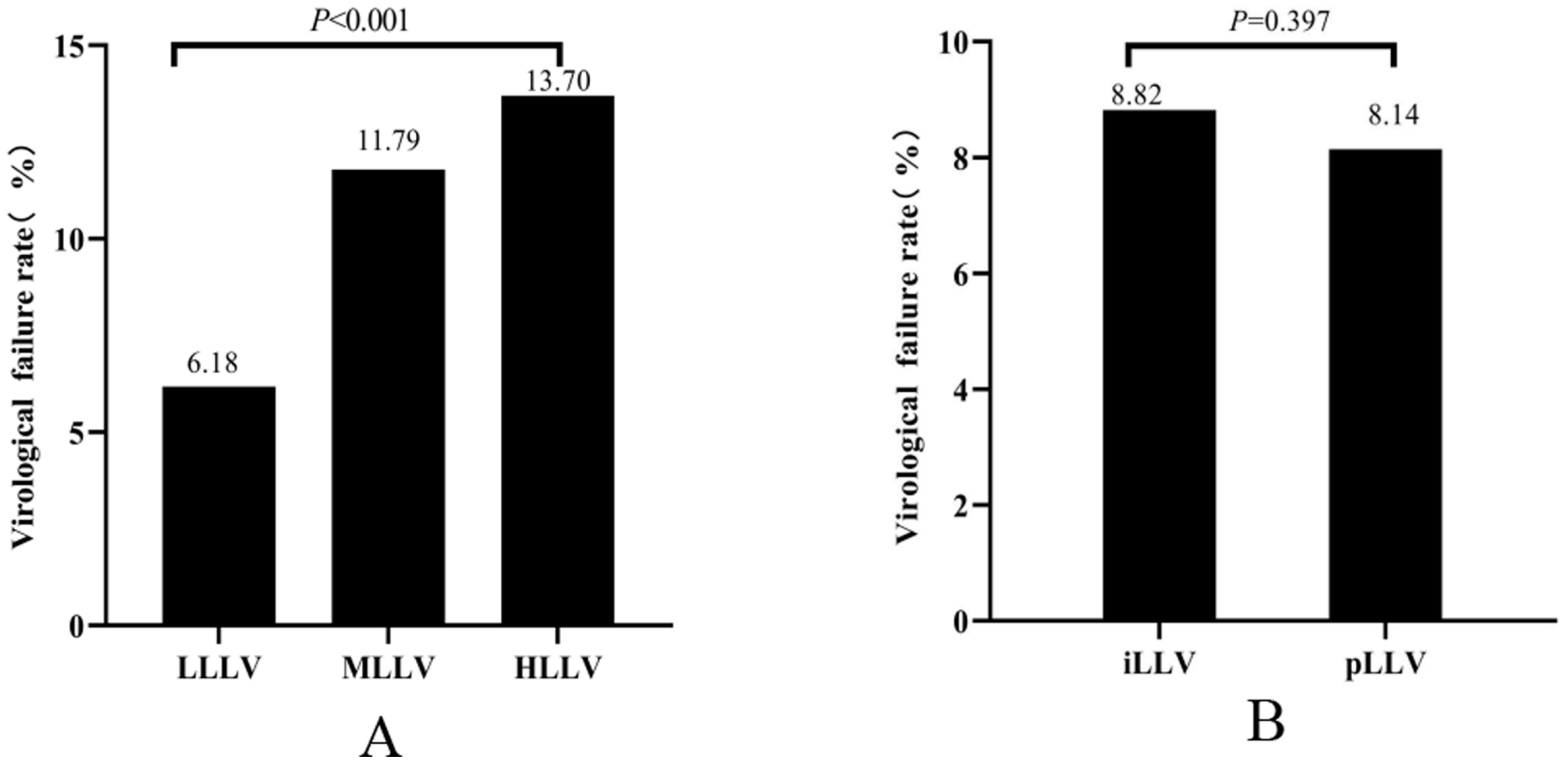
Virologic failure at different levels of LLV **(A)**. Virologic failure at different frequencies of LLV **(B)**. HLLV, high-level LLV; iLLV, intermittent low-level viremia; LLV, low-level viremia; LLLV, low-level LLV; MLLV, medium-level LLV; pLLV, persistent low-level viremia.

Of the total number of PLWHA with LLLV, 6.18% (366/5,920) experienced VF. In PLWHA with MLLV, the incidence of VF was 11.79% (221/1,875). Similarly, in PLWHA with HLLV, the incidence of VF was 13.70% (254/1,854). The incidence of VF was significantly different between the three groups (*χ^2^* = 127.848, *p* < 0.001) (Figure 2A). PLWHA with iLLV had a VF incidence rate of 8.82% (723/8,199), whereas those with pLLV had an incidence rate of 8.14% (118/1,450). However, no significant differences were observed between the iLLV and pLLV groups (*χ^2^* = 0.717, *p* = 0.397) (Figure 2B).

### 3.4 Unifactorial analysis affecting the occurrence of LLV

The results indicated that there was no significant difference between the two groups in terms of the infection route and time from confirmation to treatment (*p* > 0.05). However, significant differences were observed in the other factors (*p* < 0.05) (Table 1).

**Table 1:** Univariate analysis of the occurrence of LLV among people living with HIV/AIDS in Guizhou province of China.

					*n* (%)
Variables	Total cases (*n* = 28,613)	Non-LLV (*n* = 18,964)	LLV (*n* = 9,649)	*χ* ^2^	*p*-values
Gender				41.125	<0.001
Male	18,763 (65.58)	12,192 (64.30)	6,571 (68.10)		
Female	9,850 (34.44)	6,772 (35.71)	3,078 (31.90)		
Marriage status				11.075	0.004
Married with spouse	11,794 (41.22)	7,935 (41.84)	3,859 (40.00)		
Unmarried	5,555 (19.41)	3,684 (19.43)	1,871 (19.40)		
Divorced or widowed and unknown	11,264 (39.37)	7,345 (38.78)	3,919 (40.62)		
Education level				9.578	0.048
Illiteracy	4,057 (14.18)	2,637 (13.91)	1,420 (14.72)		
Secondary schools	11,016 (38.50)	7,295 (38.47)	3,721 (38.56)		
Junior high school	8,702 (30.41)	5,856 (30.88)	2,846 (29.68)		
High school or junior college	2,464 (8.61)	1,639 (8.64)	825 (8.55)		
College and above	2,374 (8.30)	1,537 (8.10)	837 (8.67)		
Ethnicity				15.559	0.004
Han ethnic group	20,337 (71.08)	13,423 (70.78)	6,914 (71.66)		
Miao ethnic group	2,236 (7.81)	1,526 (8.04)	710 (7.36)		
Buyei ethnic group	2,174 (7.60)	1,420 (7.49)	754 (7.81)		
Dong ethnic group	1,390 (4.86)	975 (5.14)	415 (4.30)		
Other	2,476 (8.62)	1,620 (8.54)	856 (8.87)		
Route of infection				2.289	0.515
Heterosexual transmission	25,895 (90.50)	17,186 (90.62)	8,709 (90.26)		
Homosexual transmission	2,148 (7.50)	1,395 (7.36)	753 (7.80)		
Injecting drug use and others	570 (0.20)	383 (2.02)	187 (1.94)		
Age at start of ART (years)				39.346	<0.001
18~	4,237 (14.81)	2,847 (15.01)	1,390 (14.41)		
30~	4,884 (17.07)	3,294 (17.37)	1,590 (16.48)		
40~	6,276 (21.93)	4,305 (22.70)	1,971 (20.43)		
50~	13,216 (46.19)	8,518 (44.92)	4,698 (48.69)		
Time from diagnosis to treatment (years)				4.146	0.126
<1	25,497 (89.11)	16,921 (89.23)	8,576 (88.88)		
1~	892 (3.12)	563 (2.97)	329 (3.41)		
>2	2,224 (7.77)	1,480 (7.80)	744 (7.71)		
Baseline CD4^+^ T lymphocytes (n/uL)				81.415	<0.001
<200	8,739 (30.54)	5,465 (28.82)	3,274 (33.93)		
200–350	10,052 (35.13)	6,774 (35.72)	3,278 (33.97)		
351–500	5,809 (20.30)	3,975 (20.96)	1,834 (19.01)		
>500	4,013 (14.03)	2,750 (14.50)	1,263 (13.09)		
Current treatment program					
TDF/AZT + 3TC + EFV/NVP	24,279 (84.85)	16,395 (86.45)	7,884 (8.71)	134.784	<0.001
TDF/AZT + 3TC + LPV/r	1,518 (5.31)	829 (4.37)	689 (7.14)		
Other	2,816 (9.84)	1,740 (9.18)	1,076 (11.15)		

Values in parenthesis are percentages.

ART, antiretroviral therapy; HIV/AIDS, human immunodeficiency virus/ acquired immunodeficiency syndrome; LLV, low-level viremia.

### 3.5 Multifactorial logistic regression analysis of factors related to LLV occurrence

The above seven factors with statistical significance in the single-factor analysis were included in the multivariate logistic regression model for analysis. The results showed that being female, being of the Miao or Dong ethnic group, and having baseline CD4^+^T lymphocytes > 200 cells/uL were protective factors for LLV, while being older than 50 years at the start of ART, using a second-line or other treatment regimens were risk factors for LLV (Table 2).

**Table 2:** Multifactorial logistic regression analysis of the occurrence of LLV in people living with HIV/AIDS in Guizhou province of China.

Variables	*β*	*S.E*	Wald *χ*^2^	*p*	*OR*	95% confidence interval for *OR*	
						Lower limit	Upper limit
Gender							
Male							
Female	−0.183	0.029	39.886	<0.001	0.833	0.787	0.882
Ethnicity							
Han ethnic group							
Miao ethnic group	−0.099	0.048	4.247	0.039	0.906	0.824	0.995
Buyei ethnic group	0.050	0.048	1.080	0.299	1.051	0.957	1.154
Dong ethnic group	−0.213	0.061	12.217	<0.001	0.808	0.718	0.911
Other	0.043	0.045	0.916	0.338	1.044	0.956	1.141
Age at start of ART (years)							
18~							
30~	0.009	0.049	0.035	0.852	1.009	0.916	1.112
40~	−0.047	0.053	0.788	0.375	0.954	0.860	1.058
50~	0.121	0.052	5.363	0.021	1.129	1.019	1.250
Baseline CD4^+^ T lymphocytes (n/uL)							
<200							
200–350	−0.205	0.031	43.763	<0.001	0.815	0.767	0.866
351–500	−0.243	0.036	44.721	<0.001	0.784	0.730	0.842
>500	−0.248	0.041	35.894	<0.001	0.780	0.719	0.846
Current antiretroviral treatment regimen							
TDF/AZT + 3TC + EFV/NVP							
TDF/AZT + 3TC + LPV/r	0.581	0.054	116.501	<0.001	1.787	1.608	1.986
Other	0.235	0.042	31.973	<0.001	1.265	1.166	1.372

ART, antiretroviral therapy; HIV/AIDS, human immunodeficiency virus/ acquired immunodeficiency syndrome; LLV, low-level viremia.

## 4 Discussion

The results of this study showed that the incidence of LLV among PLWHA in Guizhou Province was 33.72%. International studies have shown that the incidence of LLV in PLWHA receiving ART ranges from 15.7 to 38.7% (5, 13, 15, 16). China’s studies on the subject have reported an LLV incidence of between 3.3 and 38.7% (7, 13, 17, 18). In 2005, Guizhou Province initiated its HIV/AIDS ART program. Since 2016, the province has implemented free ART for all individuals diagnosed with HIV/AIDS, which align with national treatment standards. Concurrently, the responsibility for ART provision was transferred from the CDC to designated hospitals. However, owing to challenges such as ethnic diversity, complex geography, economic underdevelopment, and uneven distribution of medical resources, the effectiveness of ART in Guizhou Province remains inconsistent. Although ART has been proven effective in prolonging the lives of individuals and reducing morbidity and mortality, LLV can undermine treatment efficacy and lead to more severe outcomes. The relatively high incidence of LLV in Guizhou Province suggests that treatment institutions should closely monitor the VL levels of PLWHA and ensure timely assessment of their health status. Additionally, managing institutions should prioritize the follow-up care of PLWHA with LLV to prevent its occurrence and minimize the harm it causes to PLWHA.

The present study findings indicate that the occurrence of VF is related to different levels of LLV. This is consistent with the results of studies conducted in Yunnan Province (14) and Henan Province (19) in China but inconsistent with the findings of a study by Fleming et al. (20). A retrospective study in Yunnan Province, China indicated that a single LLV or two or more consecutive LLVs were associated with subsequent VF, while LLVs that occurred at intervals were not related to subsequent VF (21). Another study found that both iLLV and pLLV had an impact on the occurrence of VF, and the incidence of pLLV viral failure was higher than that of iLLV (22). The results of this study found that PLWHA in the iLLV group had a higher rate of VF than those in the pLLV group, but the difference between the two groups was not significant (*p* = 0.397). The above results suggest that more attention should be paid to the management of PLWHA with LLV in future cases of HIV antiretroviral treatment. Furthermore, the frequency of VL testing in PLWHA with LLV should be increased, and management strategies should be adjusted promptly based on VL test results. To reduce the incidence of LLV and mitigate treatment failure, more effective ART regimens should be implemented if VF occurs. Efforts to improve medication adherence among PLWHA should also be strengthened.

The results of the multifactorial research indicate that starting ART at >50 years of age is a risk factor for LLV, possibly because patients in this age group have lower immune function and weaker resistance to the virus, as they have already entered middle or older age (23). Our findings suggest that men are more prone to LLV than women, which may be related to poorer treatment adherence, greater life stress, and lower levels of self-care attention to their own health compared with women (24). Guizhou is a province with a large minority population, and differences in lifestyle and cultural beliefs exist among its various groups. This study’s findings indicate that belonging to the Miao and Dong ethnicities is associated with a lower risk of LLV, which may be related to traditional beliefs, such as nature and ancestor worship that emphasize the value of life and health. These cultural values may encourage patients to actively pursue treatment to prolong their lives. However, the underlying reasons are likely complex and require further investigation. This study also revealed that having a higher initial count of CD4^+^ T-lymphocytes was linked to a decreased likelihood of experiencing LLV. This implies that the patient’s immune system plays a crucial role in suppressing viral replication (25). Moreover, this study’s findings suggest that second-line regimens may be a risk factor for LLV. Previous studies have found that first-line regimens based on NNRTI may be more effective in suppressing VL than second-line regimens based on PI (25, 26). It may also be due to the poor health status of PLWHA or the development of resistance to first-line treatment drugs, leading to the selection of second-line treatment regimens based on PI (27).

This study has some limitations. First of all, most PLWHA in Guizhou Province have not been tested for drug resistance at the beginning of antiretroviral treatment, and drug resistance testing will be performed only when the VL test results are ≥1,000 copies/ml in the follow-up test. Therefore, at baseline, treatment regimens cannot be selected according to the drug resistance of PLWHA. Second, because the number of VL follow-up tests varied among PLWHA, PLWHA who had more follow-up tests were more likely to have LLV. Additionally, baseline VL is a key indicator for evaluating disease progression at the initiation of treatment and addressing response to subsequent treatment; however, this crucial variable was lacking in this study.

## 5 Conclusion

The findings of this retrospective cohort study indicate that the incidence of LLV among people living with HIV/AIDS treated with INSTI-sparing regimens in Guizhou Province is relatively high, which increases the risk of subsequent VF occurrence. Based on the results, we recommend intensifying detection and monitoring efforts, particularly among middle-aged and older adults living with HIV/AIDS. Public awareness and education should be enhanced, treatment adherence improved, appropriate antiretroviral therapy initiated promptly, and the occurrence of LLV effectively prevented.

## Data Availability

The original contributions presented in the study are included in the article/supplementary material, further inquiries can be directed to the corresponding author.
